# Substrate control of the electronic phase of low-buckled plumbene

**DOI:** 10.1039/d6ra06623b

**Published:** 2026-09-08

**Authors:** Niccolo Simoni, Daniel Hashemi

**Affiliations:** a Department of Physics, Optical Engineering and Nanoengineering, Rose-Hulman Institute of Technology Terre Haute Indiana 47803 USA hashemi@rose-hulman.edu

## Abstract

Low-buckled plumbene, the heaviest group-14 honeycomb monolayer with the strongest spin–orbit coupling of the family, is nevertheless a normal insulator rather than a quantum spin Hall insulator. Using first-principles calculations with a van der Waals density functional and spin–orbit coupling, we ask what a substrate does to it. On hexagonal boron nitride, whose bands stay far from the Fermi level, plumbene remains a 0.34 eV insulator. An adaptively converged first-principles Wilson loop gives *Z*_2_ = 0 for this non-centrosymmetric interface, under the same occupied-subspace protocol that reproduces nontrivial stanene and trivial free plumbene. On graphene, independent full-SOC relaxations of the hollow, top, and bridge registries all give semimetallic indirect overlaps of 0.073–0.076 eV; hollow is lower by at least 3.03 meV per cell in every cutoff and k-mesh check. Stanene acts structurally, through the buckling its strong binding forces on the sheet, and produces a robust metal within the coherent 1 × 1 models tested: the metallisation persists however the lattice mismatch is partitioned, including with plumbene unstrained, and imposing that buckling on the free sheet closes its gap on its own. Fu-Kane parity analysis, validated against stanene as a known quantum spin Hall insulator, finds free plumbene trivial at every gapped strain; compression metallises it between −3.7% and −3.9% without inverting the bands. Thus these nonmagnetic supports select ordinary-insulating, semimetallic, or metallic phases through distinct electronic and structural mechanisms, but none induces a supported quantum spin Hall phase in the commensurate models tested.

## Abstract 2

Plumbene is an insulator on hBN (0.34 eV, trivial *Z*_2_ = 0), a semimetal on graphene through electronic hybridisation, and a metal on stanene through imposed buckling: two routes to gap closure, neither of them topological.

## Graphical abstract

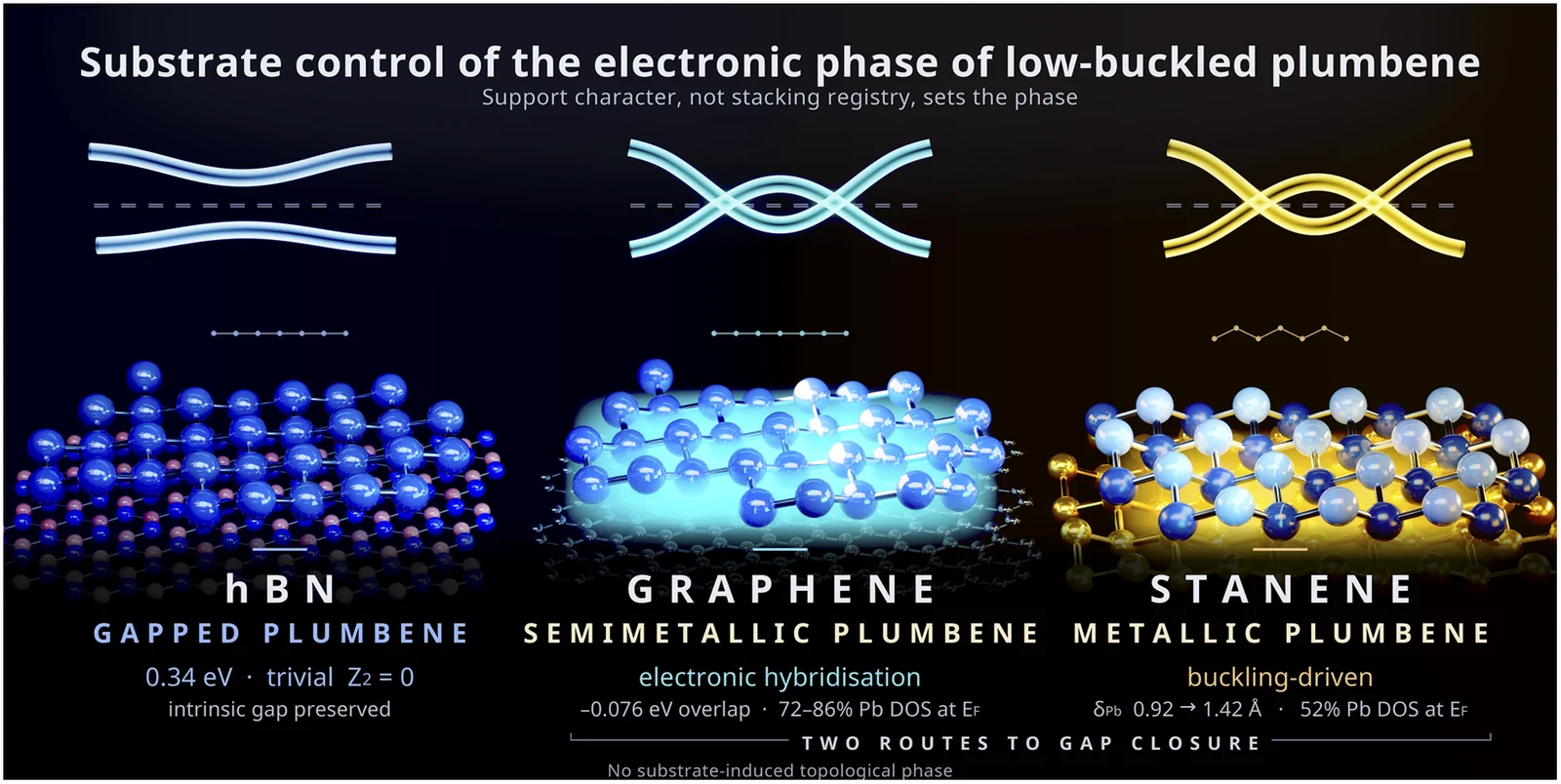

## 1. Introduction

Interest in two-dimensional honeycomb crystals of the group-14 elements grew out of a simple question left open by graphene. Graphene realises Dirac fermions but its spin–orbit gap is far too small to measure, so the quantum spin Hall state that Kane and Mele predicted for it stays hidden.^1^ Silicon, germanium, tin, and lead form the same honeycomb motif in a buckled geometry, and the strength of the spin–orbit interaction climbs sharply down the column.^2,3^ Silicene and germanene open gaps of a few to a few tens of meV,^3^ tin reaches the 0.1 eV scale,^4^ and each of these has since been grown on a supporting surface: silicene on silver,^5^ germanene on gold,^6^ and stanene on bismuth telluride.^7^

Lead sits at the bottom of the group, so plumbene should be the extreme case. Its spin–orbit splitting is the largest of the set, and the sheet has been made by segregation on a palladium-lead alloy.^8^ The surprise is that the free low-buckled plumbene monolayer is not a quantum spin Hall insulator at all. It is an ordinary band insulator with a gap near 0.4 eV.^9^ The reason is specific to lead: the very large spin–orbit coupling reorders the quadratic states at the zone centre and the Dirac states at the *K* point, and that reordering removes the band inversion that a nontrivial index needs.^9^ Plumbene also has planar and high-buckled forms with different stability and topology, reviewed recently;^21^ the low-buckled sheet studied here is a local minimum rather than the free-standing ground state, which is high-buckled. To recover a topological gap one has to push the material chemically, by adding hydrogen or halogens,^10^ by attaching amidogen, hydroxyl, or thiol groups,^11^ or by adding carriers.^9^ Each of these routes changes the sheet itself, and driven, non-equilibrium protocols can also access higher-order topological phases, including on boron nitride,^22^ a regime distinct from the equilibrium, nonmagnetic bilayers considered here.

The wider two-dimensional literature also shows how strongly topology depends on the symmetry and perturbation used. Recent examples include valley-polarised second-order phases in honeycomb ferromagnets,^23^ topology-engineered orbital Hall responses in two-dimensional ferromagnets,^24^ Floquet quantum anomalous Hall phases in altermagnets,^25^ high-Chern-number anomalous Hall phases engineered from a nonmagnetic second-order topological insulator,^26^ and nonsymmorphic protection of an antiferromagnetic topological insulator.^27^ These studies broaden the menu of accessible phases, but their magnetic, crystalline-symmetry, or driven mechanisms differ from the equilibrium, time-reversal-symmetric van der Waals proximity considered here. The comparison motivates a deliberately narrower question: how far can a nonmagnetic support move low-buckled plumbene without chemical functionalisation, magnetic order, or periodic driving?

A substrate offers a softer handle. It couples to the sheet through the van der Waals interaction and, in the best cases, leaves the intrinsic bands close to intact while still shifting the physics near the Fermi level. Substrate proximity has been used to build a high-temperature quantum spin Hall state from bismuthene on silicon carbide,^12^ and it injects a spin–orbit field into graphene whose Rashba and valley-Zeeman parts depend on how the layers register.^13^ Adsorbates can act more chemically: amidogen functionalisation changes the quantum spin Hall phase of Bi/Sb(111) films,^28^ while oxidation converts arsenene into a predicted large-gap quantum spin Hall insulator.^29^ Those cases modify the monolayer's bonding and orbital manifold, whereas the present comparison asks what is achieved by nominally non-reactive supports. For plumbene the substrate route is only lightly explored. On a ferromagnetic iron layer, combined microscopy and calculation show that plumbene hybridises strongly and, in that strongly coupled limit, develops an exchange-split gap that turns the quantum spin Hall state into a quantum anomalous Hall one.^14^ That is a chemisorbed magnetic interface rather than the weak van der Waals contacts we study here. The electronic, optical, and strain response of a plumbene sheet on boron nitride has been computed by Hiramony and co-workers,^15^ who reported, among other things, that compression closes the gap and drives the bilayer metallic. That study treated a single partner and left the topology of the bilayer aside. On the graphene side, plumbene adsorbed on and intercalated into graphene-covered SiC has been examined thermodynamically and electronically, including peeled-off graphene-plumbene bilayers,^16^ though in a supported, strained geometry rather than the free bilayer considered here. We could not find a study of plumbene on graphene, or on another Xene such as stanene, as a free van der Waals bilayer. What is open, then, is the comparison across partners of different electronic character and the topological status of the supported layer, and that is where this work is aimed.

This paper works through that gap. We take plumbene and pair it with three partners chosen to span the electronic possibilities: graphene, a semimetal with a Dirac point at the Fermi level; stanene, a heavy Xene that is itself close to a topological state; and hexagonal boron nitride, a wide-gap insulator whose bands stay away from the Fermi level. For each pair we work out how the layers prefer to stack, how strongly they bind, what happens to the plumbene bands, whether a topological index can be assigned, and whether the structure is stable. The comparison separates electronic reconstruction, substrate-imposed structural distortion, and weak gap-preserving proximity, while treating the meV-scale graphene registry ordering with explicit numerical convergence tests.

## 2. Computational details

The baseline calculations were run in VASP version 6.6.0 and the new revision-production calculations in version 6.6.1; first-principles overlap and Wannier-interface calculations used a dedicated allocation VASP 6.6.1 executable linked to Wannier90 3.1.0.^30^ All used projector augmented-wave potentials built on the PBE functional.^17^ We used the potentials that treat the Pb 5d and Sn 4d states as valence, together with the standard C, B, and N potentials. The plane-wave cutoff was 500 eV. Between 400 and 600 eV the total energy per atom drifts by a few meV (about 4.6 meV for boron nitride and 5.4 meV for graphene between the extremes); the baseline properties and initial registry screen are converged at 500 eV as documented in the ESI. Because the full-SOC Pb/graphene registry splittings are themselves on the meV scale, we separately recomputed every final SOC-relaxed geometry at 600 eV on 21 × 21 × 1 and at 500 eV on 30 × 30 × 1 meshes before assigning its ground registry. Every bilayer and every isolated slab used for a binding energy was treated with the optB86b van der Waals functional,^18^ which is needed to get the interlayer distance and binding right. The free monolayers were relaxed at the PBE level, which is the usual choice for these Xenes; because the phase we assign to the supported layer is a qualitative property of the band character at the Fermi level rather than a fine gap value, it does not depend on the small differences between the two functionals. We checked this directly by recomputing the boron nitride bilayer, the one insulating heterostructure, with plain PBE at the optB86b geometry: the gap is 0.345 eV, against 0.34 eV with optB86b, so the insulating phase does not depend on which of the two functionals is used. A further check points the same way: the free monolayer held at each substrate strain stays gapped (Section 3.6). To test functional robustness directly at the relativistic level, we also performed self-consistent HSE06 + SOC calculations^33^ for free plumbene and all three bilayers at a 500 eV cutoff on Gamma-centred 6 × 6 × 1 meshes, which contain *K*; the results are reported in Sections 3.1 and 3.5 and ESI S6.

Reciprocal space was sampled on Gamma-centred grids whose divisions are multiples of three, so that the hexagonal *K* point falls on the mesh. This detail matters: a grid that misses K produces a spurious gap of about 1 eV in graphene, and sampling K removes it. The self-consistent grid was 15 × 15 × 1; band gaps and band extrema were extracted from a denser 21 × 21 × 1 grid; non-spin–orbit densities of states used 30 × 30 × 1 and the spin–orbit layer-resolved densities of states used 21 × 21 × 1 (with convergence checks at 27 × 27 × 1 and 33 × 33 × 1, ESI S7); band structures were traced along K to Gamma to M to K with K at (2/3, 1/3, 0). We used Gaussian smearing with a 0.05 eV width for the semiconducting cases. For the metallic bilayers the self-consistent step used Methfessel-Paxton smearing, which converges the charge density where Gaussian smearing stalls. Energies were converged to 10^−6^ eV, and the baseline geometries were relaxed until every force fell below 0.01 eV Å^−1^. Because the initial non-SOC registry comparison suggested geometry sensitivity, every graphene registry was re-relaxed independently with SOC to a stricter 0.001 eV Å^−1^ threshold and 10^−7^ eV electronic tolerance on the 15 × 15 × 1 grid, then evaluated with SOC on the 21 × 21 × 1 grid. Each cell carries at least 20 Å of vacuum. For the asymmetric slabs a dipole correction was applied in the static electrostatic calculations from which the two-sided work functions and planar-averaged potentials are taken; it was not applied during the geometry relaxations, whose forces are insensitive to it at these vacuum sizes.

Spin–orbit coupling was included in the noncollinear formalism with the spin quantization axis along *z*. The monolayer lattice constants were obtained from a variable-cell relaxation in which the vacuum vector also participates; because the out-of-plane stress of an isolated slab with more than 20 Å of vacuum is negligible, the vacuum axis changed by less than 0.6% and remained above 20 Å, and a scan of the in-plane lattice constant at fixed vacuum confirms the same in-plane optimum. Under strain the cell was fixed and the internal coordinates relaxed. The bilayers were built on fixed commensurate cells; the strain carried by each layer is given in Table 2. The HSE06 + SOC calculations used the standard 25% short-range exact-exchange fraction and a 0.2 Å^−1^ screening parameter at the PBE + SOC-relaxed geometries.

The *Z*_2_ index of the gapped, centrosymmetric systems (the free monolayer and its strained forms) was obtained from the Fu-Kane parity criterion:^19^ the product of the inversion parities of the occupied states at the four time-reversal-invariant momenta. For each system the spin–orbit charge density was first converged self-consistently on a full-zone 15 × 15 mesh; the four time-reversal-invariant momenta were then computed non-self-consistently from that fixed density, and the parities were read from the resulting wavefunctions with the irrep package,^20^ after shifting the cell so the inversion centre sits at the origin. We validated the whole procedure on the stanene monolayer, a known quantum spin Hall insulator, for which it returns the nontrivial index. Because Pb/hBN lacks inversion, Z2Pack^31^ evaluated its hybrid Wannier charge centres as the eigenphases of closed Wilson loops built directly from first-principles overlap matrices for all 60 occupied spinor bands. VASP wrote the occupied-subspace overlaps through the Wannier90 interface, but no fitted or disentangled tight-binding Hamiltonian enters the index. The calculation adaptively refined both the points on each closed loop and the number of transverse loops until the position, largest-gap, and motion criteria all converged. The identical occupied-subspace workflow was required to reproduce the stanene (*Z*_2_ = 1) and plumbene (*Z*_2_ = 0) parity controls before Pb/hBN was accepted.

To separate hybridisation from structural distortion we used a frozen-geometry control, computing the isolated plumbene layer at the exact coordinates and cell it takes in each relaxed bilayer, and we repeated the stanene case at several imposed common lattice constants (all coherent 1 × 1 cells, so that the same lattice mismatch is partitioned differently between the two layers). Charge transfer was obtained from a Bader partition of the all-electron density, reconstructed from the core and valence contributions, and the interfacial charge redistribution from the difference between the bilayer density and its isolated layers. Work functions were taken from the planar-averaged electrostatic potential on each side of the slab separately, since the asymmetric bilayers have two inequivalent vacuum levels. Phonons were obtained from finite displacements and post-processed in Phonopy^32^ with space-group force-constant symmetrisation and the translational acoustic sum rule; residual soft-mode eigenvectors were analysed explicitly. Room-temperature behaviour was tested with Born-Oppenheimer molecular dynamics in the canonical ensemble. Each unit cell was first re-relaxed below 0.001 eV Å^−1^, then expanded exactly to a 4 × 4 free-plumbene sheet or a 3 × 3 bilayer. Trajectories used PREC = Normal, a 500 eV cutoff, EDIFF = 10^−5^ eV, Gaussian smearing with *σ* = 0.05 eV, real-space projection, and the blocked-Davidson/RMM-DIIS solver (ALGO = Fast); symmetry was switched off. Free plumbene and Pb/stanene used a 200-iteration electronic ceiling with default mixing and band counts. Pb/graphene and Pb/hBN required solver-robustness measures that cannot change the converged physics, only whether it is reached: damped linear mixing (AMIX = 0.1, BMIX = 10^−4^, AMIN = 0.01), a 500-iteration ceiling, and NBANDS = 312 (resolved to 320 by band-group parallelism). These were adopted after initial production attempts on both systems failed with an identical RMM-DIIS subspace-diagonalisation (ZHEGV) collapse localised in the highest empty bands; removing those bands eliminated the failure and both accepted re-runs converged at every ionic step. Before any production run each system was screened with paired 48-step trials under two frozen velocity seeds, and a production trajectory was started only where both seeds converged at every ionic step. Each accepted trajectory is a single uninterrupted 1500-step (3 ps) run at 300 K with a 2 fs time step and a Nosé-Hoover thermostat, started from ionic step 1; no screening trajectory or partial segment was reused or concatenated. Acceptance required the electronic loop to reach EDIFF at every one of the 1500 ionic steps, with standard output, OUTCAR, and scheduler standard error checked for electronic non-convergence or internal VASP failure; any trajectory failing these was rejected in full. In-plane elastic constants were fitted from biaxial and Cartesian-uniaxial energy–strain curves over −2% to +2%, with internal coordinates relaxed below 0.001 eV Å^−1^ at every strain.

## 3. Results and discussion

### 3.1 Monolayers as a baseline

Table 1 lists the relaxed geometry and the gap of each monolayer we use later, taken straight from the output files. The lattice constants land within 0.3% of the accepted values and the bucklings within 0.01 Å, so the setup is sound. Two entries anchor the rest of the work. Graphene comes out as a Dirac semimetal with no gap at K once K is on the grid, with and without spin–orbit coupling. Plumbene opens a gap when spin–orbit coupling is switched on, starting from a nearly closed PBE spectrum: on the relaxed sheet (buckling 0.923 Å) the valence and conduction band extrema both sit at K, giving a 0.42 eV gap. That gap belongs to a normal insulator, and Section 3.6 confirms its trivial index, so it is the reference we compare the bilayers against rather than a topological gap. On the same geometry a self-consistent HSE06 + SOC calculation gives a direct and fundamental *K*-point gap of 0.581 eV on a Gamma-centred 6 × 6 × 1 mesh. The hybrid result supports the robustness of the gapped phase and gap scale; the *Z*_2_ index itself is obtained from the occupied-state topology in Section 3.6. The 0.42 eV PBE + SOC gap agrees with published values for the low-buckled sheet.^9,21^

**Table 1:** Relaxed lattice constant, nearest-neighbour bond, buckling, and band gap of the monolayers, without and with spin–orbit coupling

Monolayer	*a* (Å)	Bond (Å)	Buckling *δ* (Å)	Gap PBE (eV)	Gap PBE + SOC (eV)
Plumbene	4.937	2.996	0.923	0 (near-closed)	0.42 (at K)
Stanene	4.674	2.829	0.850	0 (Dirac)	0.073
Silicene	3.867	2.278	0.452	0 (Dirac)	0.0015 (1.5 meV)
hBN	2.513	1.451	0.000	4.66	4.66
Graphene	2.468	1.425	0.000	0 (Dirac)	0

### 3.2 Commensurate cells

**Fig. 1:**
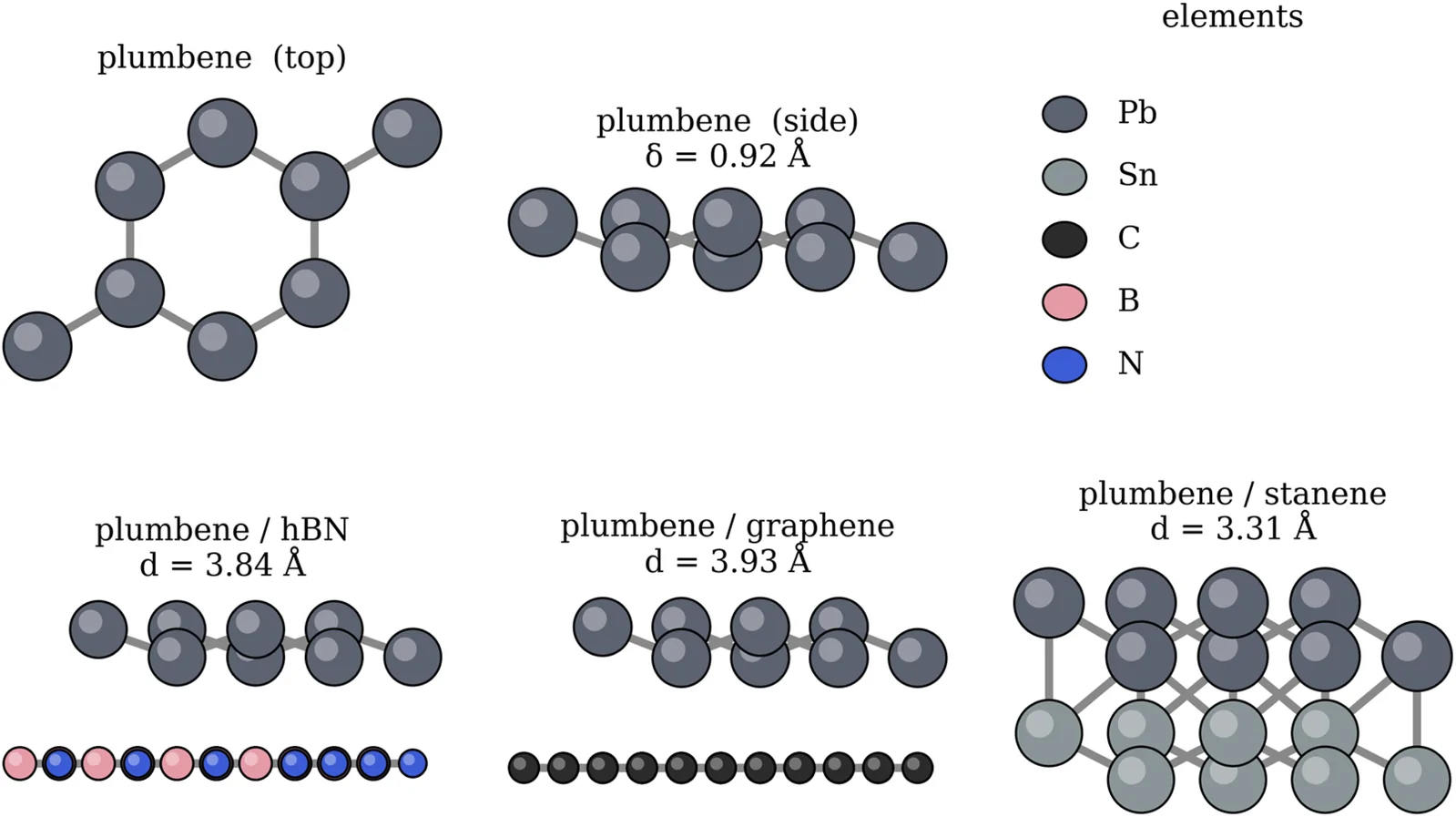
Relaxed structures. Top: the plumbene monolayer in top and side view; the honeycomb is buckled by 0.92 Å. Bottom: side views of the three bilayers with their interlayer separations. Pb dark grey, Sn grey-green, C black, B pink, N blue.

Each bilayer was built on a small commensurate cell so that both layers keep a periodic registry (Fig. 1). Table 2 lists the cell, the supercell match, and the strain each layer carries. Plumbene keeps its own lattice on graphene almost exactly, is stretched by 1.8% on boron nitride, and is compressed by 2.8% on stanene, while the partner takes up the complementary strain. The lattice mismatch with stanene is the largest, about 5.6%, and Section 3.5 shows the stanene result does not depend on how that mismatch is partitioned between the layers.

**Table 2:** Commensurate cells and the biaxial strain carried by each layer

Bilayer	*a* (Å)	Supercell match	Plumbene strain	Partner strain
Pb/graphene	4.936	1 × 1 Pb/2 × 2C	−0.0%	+0.0%
Pb/hBN	5.024	1 × 1 Pb/2 × 2 hBN	+1.8%	−0.0%
Pb/stanene	4.800	1 × 1 Pb/1 × 1 Sn	−2.8%	+2.7%

### 3.3 How the layers stack

Before fixing a bilayer we scanned the registries of each pair and relaxed them. Table 3 gives the energy of each registry relative to the lowest. On stanene, plumbene settles firmly into the AA arrangement, with the bridge and AB registries lying 505 and 719 meV per cell higher. On boron nitride the registries fall within about 16 meV; the closest pair (hollow and over-N) differs by only 1.3 to 1.6 meV across our convergence checks, which at a 0.01 eV Å^−1^ force tolerance means the two are effectively degenerate rather than uniquely ordered. Establishing genuinely free sliding would need a continuous sliding path or a registry map. Because the initial mixed-protocol graphene comparison appeared sensitive to sub-ångström structural changes, we use only the stricter full-SOC relaxations and matched SOC statics to establish both registry ordering and phase. All three independent protocols select hollow: its margin over the next registry is 3.045 meV per cell at 500 eV/21 × 21 × 1, 3.028 meV at 600 eV/21 × 21 × 1, and 3.275 meV at 500 eV/30 × 30 × 1. At the production setting, bridge and top are 3.045 and 3.060 meV per cell above hollow and are not meaningfully ordered relative to each other. Crucially, all three SOC-relaxed registries are semimetallic indirect-overlap states, so the revised result is not a registry-driven metal–insulator switch.

**Table 3:** Energy of each stacking registry relative to the ground-state registry of the same bilayer

Bilayer	Registry	Δ*E* (meV per cell)
Pb/stanene	AA (ground state)	0
Pb/stanene	Bridge	505
Pb/stanene	AB	719
Pb/graphene	Hollow (ground state)	0.000
Pb/graphene	Bridge	3.045
Pb/graphene	Top	3.060
Pb/hBN	Hollow (ground state)	0
Pb/hBN	N-top	2
Pb/hBN	B-top	16

### 3.4 Binding

Binding energies appear in Table 4, defined as the bilayer energy minus the two isolated slabs computed in the same commensurate cell and strain state. The accepted hollow Pb/graphene ground binds by −0.331 eV per cell (−0.165 eV per Pb, −15.7 meV Å^−2^) at a mean-plane separation of 3.93 Å. The boron nitride values sit in the physisorption window; the stanene interface, bound roughly four times more strongly, is better described as a strongly coupled van der Waals interface. The Pb–Sn AA pair binds most strongly at −62 meV Å^−12^, which fits its heavy crystalline partner and its firm registry. The interlayer separations do not simply track the binding strength: the AA stanene pair is far more strongly bound than the AB one yet relaxes to a slightly larger separation, so the two coordinates are not interchangeable.

**Table 4:** Binding energies and interlayer separations of the bilayers. The interlayer distance is defined throughout as the separation between the mean plane of the lead atoms and the mean plane of the partner atoms, measured on the relaxed geometry used for the corresponding binding calculation

Bilayer	*E*_b_ (eV/cell)	eV/Pb	meV Å^−12^	Interlayer distance (Å)
Pb/stanene (AA)	−1.234	−0.617	−61.9	3.31
Pb/stanene (AB)	−0.516	−0.258	−25.8	3.24
Pb/graphene (hollow)	−0.331	−0.165	−15.7	3.93
Pb/hBN (AB)	−0.346	−0.173	−15.8	3.91
Pb/hBN (AA)	−0.331	−0.165	−15.1	3.93

### 3.5 The substrate sets the phase

**Fig. 2:**
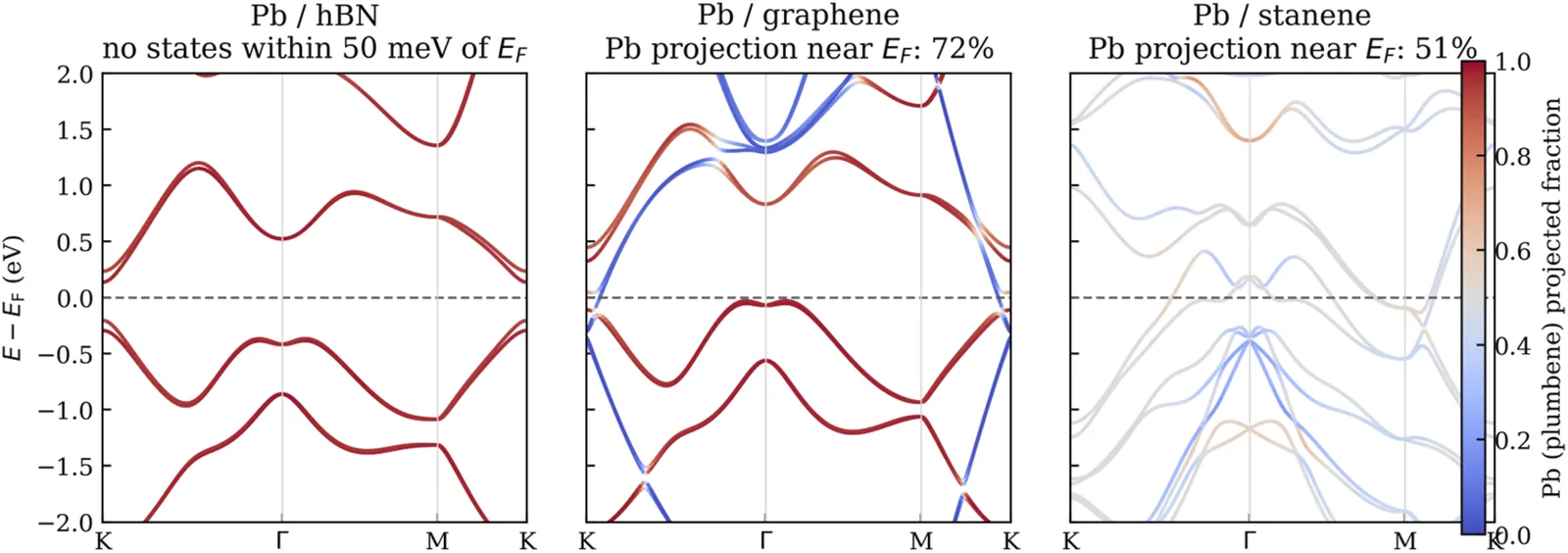
Layer-projected spin–orbit band structures of the three bilayers, coloured by lead (plumbene) character from blue for the partner to red for plumbene. On boron nitride the plumbene-derived bands hold a 0.34 eV gap, while on stanene lead-derived bands cross the Fermi level decisively. The accepted hollow Pb/graphene ground is semimetallic through a −0.076 eV indirect overlap while retaining a positive 0.197 eV minimum direct gap.

The three partners perturb plumbene in sharply different ways. Boron nitride, whose bands stay away from the Fermi level, preserves the gap, while stanene closes it through a substrate-imposed structural distortion. Graphene follows a third route: the accepted hollow interface is a nonmagnetic semimetal with a −0.076 eV indirect overlap but a positive 0.197 eV minimum direct gap, and 72.4% of the band-path states within 50 meV of *E*_F_ are projected onto Pb (Fig. 2).

On hexagonal boron nitride the plumbene sheet stays a gapped insulator. The number of filled bands is the same at every *k* point, and a 0.34 eV gap runs across the whole zone. Boron nitride keeps its bands several eV away from the Fermi level, so it adds no states there, and the intrinsic plumbene gap survives almost unchanged.

On stanene the bands are driven deep through the Fermi level: the overlap is several tenths of an eV and the metallic phase is robust to every control we apply below. On boron nitride the density of states at the Fermi level is zero because the plumbene-derived 0.34 eV gap remains intact. The accepted hollow Pb/graphene ground has a −0.076 eV fundamental gap (an indirect band overlap) while retaining a positive 0.197 eV minimum direct gap. Top and bridge give the same qualitative phase, with −0.073 and −0.074 eV indirect overlaps. Thus the full-SOC registry calculation places Pb/graphene on the semimetallic side for every candidate stacking rather than at a stacking-selective metal–insulator boundary. The stanene layers contribute comparably at the Fermi level (a lead share near 52%). We report these as density-of-states weights, not transport claims, since density of states is not conductivity.

**Fig. 3:**
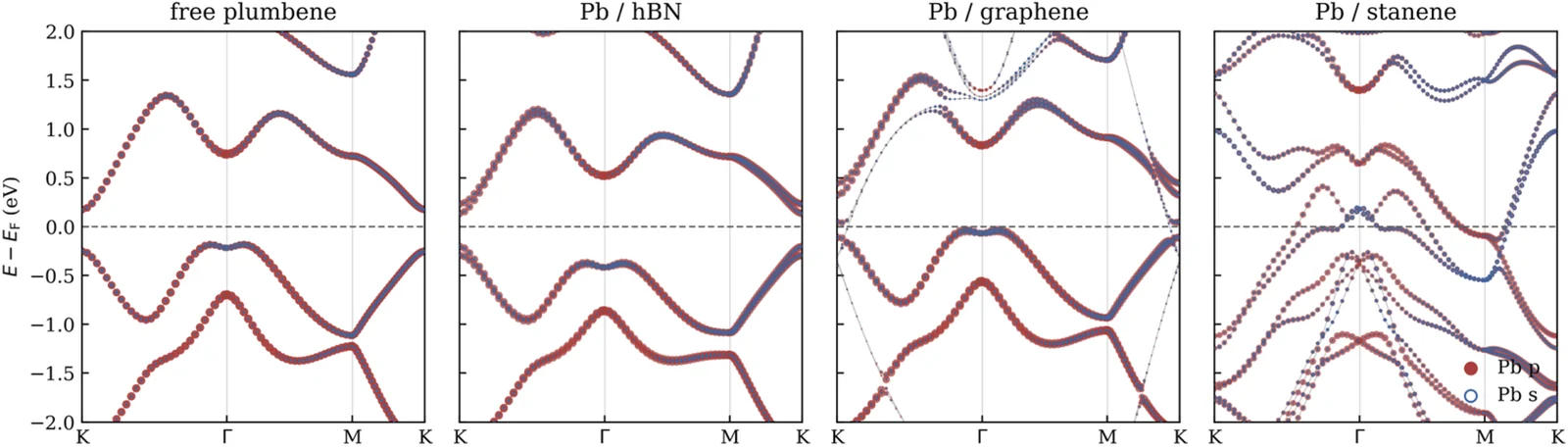
Pb-orbital-resolved PBE + SOC bands for free plumbene and the three bilayers. Filled red markers give total Pb p weight and open blue markers give Pb s weight; thin grey lines show the underlying bands. The low-energy plumbene-derived states are predominantly p-like.

The orbital projection in Fig. 3 separates the Pb s contribution from the summed Pb p contribution on the same spin–orbit band structures. The band edges of free plumbene are overwhelmingly Pb-p-derived, and this remains the organizing orbital character in all three bilayers. Boron nitride leaves an isolated Pb-p gap. Graphene introduces its Dirac-derived manifold and redistributes Pb-p weight close to the Fermi level; its final phase and isolated-layer control are evaluated on the validated SOC ground registry below. Stanene contributes a second heavy-element manifold and mixes with the Pb-p bands after the large substrate-induced buckling. The Pb-s contribution lies mainly farther from the Fermi level. Thus the substrate response is not a simple exchange of Pb s and p band edges; it is a partner-dependent addition and hybridisation of states acting on a low-energy Pb-p manifold.

The completed hybrid-functional checks strengthen all four phase assignments. HSE06 + SOC increases the free-plumbene gap from 0.418 to 0.581 eV and the Pb/hBN gap from 0.34 to 0.492 eV, while Pb/stanene retains a large indirect band overlap of −0.554 eV despite a positive 0.381 eV minimum direct gap. Thus hBN remains insulating and the gapless Pb/stanene phase persists under HSE06 + SOC. For the accepted hollow Pb/graphene ground, a fresh symmetry-preserving calculation with zero initial moments converges to a maximum site moment of 0.0 *µ*_B_ and retains an indirect overlap of −0.056 eV; its 0.0008 eV minimum direct gap is at the resolution of the hybrid mesh. This independently confirms a nonmagnetic semimetal rather than a PBE-specific or spin-polarized artefact. These calculations use the same 6 × 6 × 1 mesh containing K; because this mesh is coarser than the production PBE + SOC sampling, we use it to test qualitative phase and gap robustness rather than to replace the dense-mesh extrema.

The metallisation could come either from the partner's states or from the way the substrate distorts the plumbene sheet, and the two must be separated. We do this with a frozen-geometry control: the plumbene layer is taken out of each relaxed bilayer with its exact atomic positions and cell kept fixed, and computed in isolation, with no partner present. For the accepted hollow Pb/graphene ground, the full interface has a −0.076 eV indirect overlap, whereas the isolated Pb layer frozen at the same 0.97 Å buckling is gapped by 0.375 eV (minimum direct gap 0.428 eV). The gap collapse therefore requires the graphene interface and is an electronic-reconstruction/hybridisation effect rather than a geometry-only metallisation. On boron nitride the extracted layer keeps a 0.43 eV gap, in line with the intact bilayer gap. Stanene behaves differently. Its strong binding drives the plumbene buckling from 0.92 to 1.42 Å, and the extracted layer at that distorted geometry is already metallic on its own, with the fundamental gap closed even without the stanene present.

Two further controls show this structural route is neither sensitive to how the lattice mismatch is shared between the layers nor loosely attributed to buckling. First, the outcome does not depend on the strain partitioning: computing Pb/stanene in coherent 1 × 1 cells with the common lattice fixed at plumbene's own value, so that the lead layer carries no strain, still gives a buckling of 1.28 Å and a metallic layer, and the same holds at stanene's lattice and at the energy-optimised cell. The stanene interface distorts and metallises the lead sheet whether or not plumbene is compressed. These are all coherent 1 × 1 models; the 5.6% mismatch cannot be relieved in a small cell, so we do not test a larger coincidence supercell or a rotated moiré, and we restrict the claim to the strain-partitioning it does probe. Second, buckling alone is sufficient: imposing a 1.42 Å buckling on the free, unstrained sheet, with nothing else changed, closes its gap (a fundamental overlap of about −0.5 eV), whereas the equilibrium 0.92 Å buckling reproduces the 0.42 eV insulator. The chain is then clear: the stanene contact forces a large buckling, and that buckling on its own closes the gap. Graphene is the complementary control: its frozen Pb sheet remains a 0.375 eV insulator, so the interface semimetal arises from electronic reconstruction with graphene, whereas the stanene-distorted Pb sheet is metallic even after the partner is removed.

### 3.6 Topology and its stubbornness

A *Z*_2_ index is well defined for the gapped systems. The free monolayer and its strained forms keep the inversion symmetry of the buckled honeycomb, so their index follows from the parities of the occupied states at the four time-reversal-invariant momenta, the criterion of Fu and Kane.^19^ We read these parities from the wavefunctions with the irrep package.^20^ To make the procedure trustworthy we first run it on stanene, a monolayer that is an established quantum spin Hall insulator. It returns *Z*_2_ = 1: the number of parity-odd occupied states is 12 (six Kramers pairs) at the zone centre and 14 (seven pairs) at each of the three M points, giving parity products *δ* = +1 at *Γ* and *δ* = −1 at each M; the product over the four momenta is −1, the fingerprint of the band inversion. Free plumbene, computed in exactly the same way, returns *Z*_2_ = 0, with 14 parity-odd states (seven pairs, *δ* = −1) at every one of the four momenta, so the product is +1. The two materials differ only at the zone centre, which is precisely where plumbene's strong spin–orbit coupling reorders the band edges and removes the inversion that stanene keeps. Plumbene is therefore an ordinary insulator, and the classification comes from a method validated here on a known nontrivial case.

**Fig. 4:**
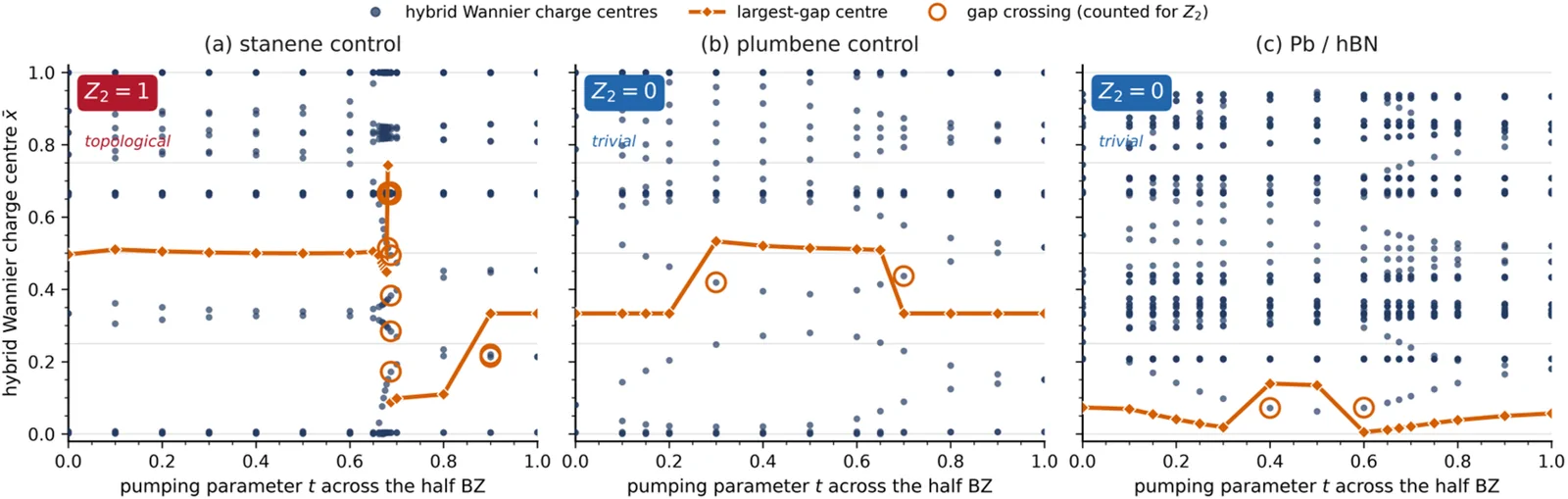
Adaptively converged first-principles Wilson loops under the identical occupied-subspace protocol for (a) the stanene positive control, (b) the plumbene negative control, and (c) non-centrosymmetric Pb/hBN. Open black circles are the hybrid Wannier charge centres and blue diamonds mark the centre of the largest gap on each transverse line. The controls return *Z*_2_ = 1 and 0, respectively, while Pb/hBN returns *Z*_2_ = 0.

Boron nitride breaks inversion symmetry, so parity cannot classify the Pb/hBN bilayer. We therefore evaluate the hybrid Wannier charge-centre evolution directly from the first-principles occupied-subspace Wilson loop, after requiring the identical workflow to reproduce the known stanene (*Z*_2_ = 1) and plumbene (*Z*_2_ = 0) controls (Fig. 4). The Pb/hBN calculation contains 60 charge centres on each of 16 adaptively selected transverse lines. Every position, largest-gap, and motion convergence check passes, and the maximum endpoint Kramers-pair splitting is 8.26 × 10^−9^. The largest-gap reference is crossed twice, an even parity that gives *Z*_2_ = 0. This direct result establishes that supported plumbene on hBN is topologically trivial. The older five-separation gap scan is retained in ESI S10 only as supporting evidence about one adiabatic path; it is not used to determine the index.

We then asked whether strain could do what proximity cannot. Table 5 follows the fundamental gap of free plumbene across biaxial strain, with the band extrema and their locations extracted from a dense mesh. Under tension the gap widens, staying a direct gap at K, and the index stays trivial. Under compression the valence-band maximum moves off K toward the zone centre and the fundamental gap closes as an indirect Γ–K overlap. A dense strain series through the transition region (−3.5% to −4% in 0.1% steps) brackets the crossing between −3.7% (a 0.010 eV gap) and −3.9% (a 0.024 eV overlap); the value at −3.8% is a 4 meV overlap, within our numerical resolution of a few meV, so we quote the transition as lying between −3.7% and −3.9% rather than as a precise critical strain. Beyond it the sheet is a semimetal and no index is assigned. Wherever a gap remains, from −3.5% to +8%, the parity calculation returns *Z*_2_ = 0. This corrects a point that a direct gap at K alone would hide, since that direct gap stays near 0.42 eV even where the global gap has closed. The compression-driven closing is a plain metallisation, the same effect Hiramony and co-workers saw for plumbene on boron nitride under pressure,^15^ not a band inversion: the gap does not reopen with switched parities. Plumbene stays a trivial insulator under every mechanical handle that leaves it gapped.

**Table 5:** Fundamental gap, band-edge locations, and *Z*_2_ index of free plumbene under biaxial strain (internal coordinates relaxed at each fixed cell). Compression closes the gap as an indirect *Γ*–K overlap and metallises the sheet between −3.7% and −3.9% (the −3.8% value is within numerical resolution of zero); where a gap remains the index is trivial

Biaxial strain	VBM (k)	CBM (k)	Fundamental gap (eV)	*Z*_2_
−8%	*Γ*	K	Metallic (−1.21)	n/a
−6%	*Γ*	K	Metallic (−0.54)	n/a
−4%	*Γ*	K	Metallic (−0.04)	n/a
−3.9%	*Γ*	K	Metallic (−0.02)	n/a
−3.8%	Near *Γ*	K	−0.004	n/a
−3.7%	Near *Γ*	K	0.010	0
−3.5%	Near *Γ*	K	0.036	0
−3%	Near *Γ*	K	0.134	0
−2.5%	Near *Γ*	K	0.221	0
−2%	Near *Γ*	K	0.279	0
0%	K	K	0.418	0
+2%	K	K	0.419	0
+4%	K	K	0.424	0
+6%	K	K	0.430	0
+8%	K	K	0.439	0

### 3.7 Charge transfer, work function, and stability

Table 6 collects the interface quantities. Bader analysis, referenced to the all-electron core and valence densities, gives the charge that moves across each interface; a positive value means electrons gained by the plumbene layer. The work function needs two numbers because the asymmetric slabs have different vacuum levels on their two faces. For hollow Pb/graphene, the Pb layer gains −0.027 e per cell and the Pb- and graphene-face work functions are 4.40 and 4.12 eV, respectively. On stanene the lead sheet gains about 0.02 e and the two work functions are both about 4.42 eV. Across Pb/hBN the lead sheet loses about 0.03 e and the two vacuum levels differ by 0.38 eV. Because Pb/hBN is insulating, its Fermi level sits inside a gap; the more transferable quantities are the ionisation potential (vacuum level minus valence-band maximum) of 4.19 eV and the electron affinity (vacuum level minus conduction-band minimum) of 3.85 eV on the plumbene face, which bracket the gap.

**Table 6:** Bader charge on the plumbene layer (positive = gained) and two-sided work function of each interface

Interface	Charge on Pb layer (e/cell)	Work function, Pb face/partner face (eV)
Pb/graphene	−0.027	4.40/4.12
Pb/hBN	−0.03	3.85/4.23
Pb/stanene	+0.02	4.42/4.42

**Fig. 5:**
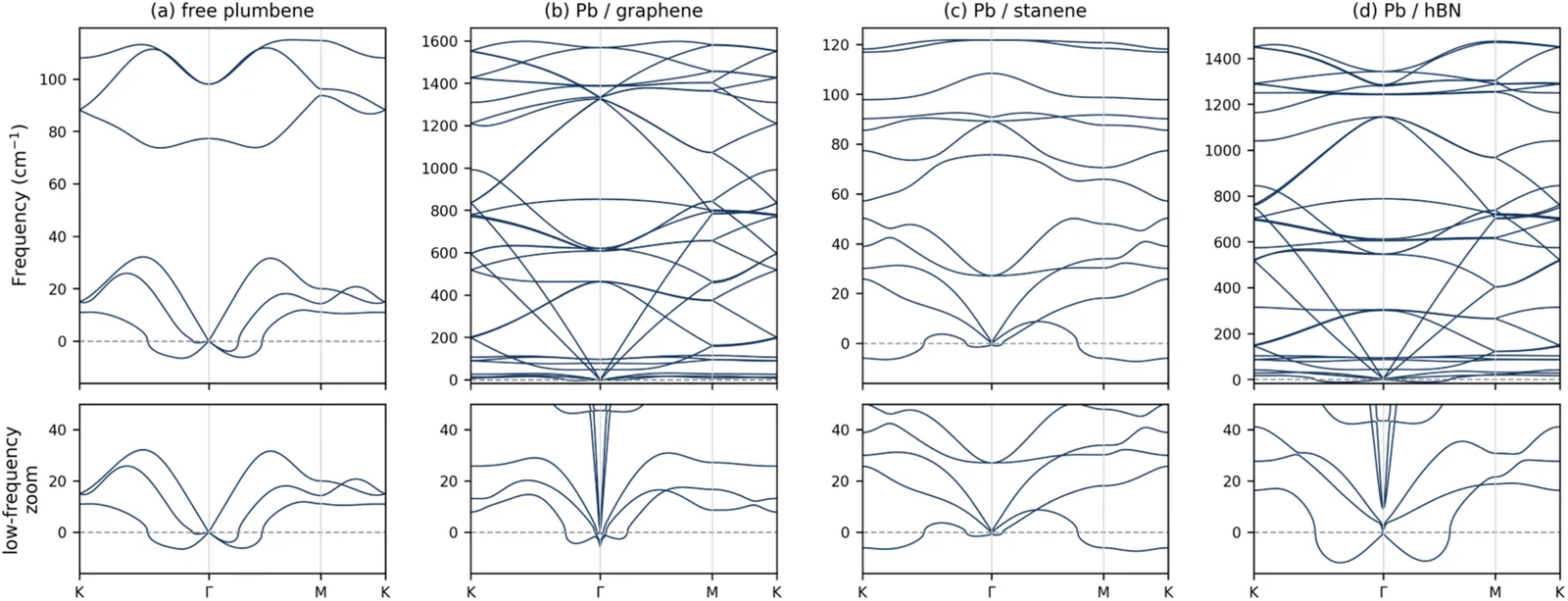
Phonon dispersions after force-constant symmetrisation and enforcement of the translational acoustic sum rule. The lower row magnifies the low-frequency window. Residual minima remain below 12.1 cm^−1^ in magnitude and are analysed by eigenvector in the text and ESI rather than being uniformly assigned to a flexural artefact.

Lattice dynamics are shown in Fig. 5. Finite-displacement force constants were computed without SOC on a 4 × 4 supercell for free plumbene, 3 × 3 supercells for the graphene and stanene bilayers, and a 2 × 2 supercell for the ten-atom hBN pair. We enforced the translational acoustic sum rule by space-group and traditional force-constant symmetrisation; the maximum force-constant drift after this treatment is zero to the printed precision. Small negative frequencies persist, with minima of −6.55, −5.66, −7.35, and −11.85 cm^−1^ for free plumbene, Pb/graphene, Pb/stanene, and Pb/hBN, respectively. The Pb/graphene minimum occurs at Gamma and is essentially an in-plane counter-motion of the two layers (out-of-plane fraction 7.3 × 10^−6^; interlayer in-plane eigenvector cosine −1.000), identifying it as a weak interlayer shear mode rather than a flexural mode. Its small magnitude is consistent with the shallow registry-energy landscape of this weakly bound interface, but we retain it as a residual soft mode rather than treating that interpretation as proof of stability. Their eigenvectors are not all the same. The free-plumbene and Pb/hBN minima are 81% and 80% out-of-plane, consistent with the well-known numerical sensitivity of the quadratic flexural branch to finite-supercell truncation. The Pb/stanene minimum is a small in-plane acoustic-like feature away from Gamma. ASR therefore removes translational drift but does not license us to relabel every residual negative mode as a flexural artefact. We retain the negative values, report their mode character, and interpret them as soft modes whose magnitude and supercell convergence limit any claim of harmonic dynamical stability. Independent energy–strain fits give C11 = 17.15 N m^−1^, C12 = 11.10 N m^−1^, and C66 = 3.03 N m^−1^; C11 > |C12| and C66 > 0 satisfy the two-dimensional Born criteria for infinitesimal in-plane elastic stability of the low-buckled local minimum. The complementary finite-temperature evidence is four fresh 1500-step (3.0 ps) trajectories completed, of which free plumbene retained every initial bond while Pb/graphene, Pb/stanene and Pb/hBN underwent Pb–Pb bond rearrangement (minimum retained fraction 0.815), with mean temperatures of 294.9–299.9 K, a maximum absolute extended-system-energy drift of 1.086 meV atom^−1^ ps^−1^, and a minimum bilayer mean-plane-separation ratio of 79.1%. These results are short finite-cell 300 K tests, not evidence of macroscopic or long-time thermodynamic stability.

## 4. Conclusions

The nonmagnetic substrate is a decisive control parameter for low-buckled plumbene. Full-SOC relaxation and three matched convergence protocols select hollow Pb/graphene as the ground registry; it is a nonmagnetic semimetal with a −0.076 eV indirect overlap and a positive 0.197 eV minimum direct gap. Its frozen Pb layer instead has a 0.375 eV gap, identifying electronic reconstruction with graphene. Pb/stanene follows a structural route because its distorted Pb layer remains metallic without stanene, whereas hBN preserves an insulating Pb-p manifold. HSE06+SOC retains these qualitative phase assignments, and direct first-principles Wilson loops give *Z*_2_ = 0 for non-centrosymmetric Pb/hBN while reproducing the stanene/plumbene *Z*_2_ = 1/0 controls.

ASR-treated phonons retain explicitly reported soft modes rather than being declared uniformly stable, and the elastic constants satisfy the two-dimensional Born criteria. Complementary AIMD gives four fresh 1500-step (3.0 ps) trajectories completed, of which free plumbene retained every initial bond while Pb/graphene, Pb/stanene and Pb/hBN underwent Pb–Pb bond rearrangement (minimum retained fraction 0.815), with mean temperatures of 294.9–299.9 K, a maximum absolute extended-system-energy drift of 1.086 meV atom^−1^ ps^−1^, and a minimum bilayer mean-plane-separation ratio of 79.1%. These results are short finite-cell 300 K tests, not evidence of macroscopic or long-time thermodynamic stability. Thus the combined evidence supports substrate-specific electronic and structural mechanisms, while deliberately stopping short of claims about experimental synthesizability or long-time stability.

## Conflicts of interest

There are no conflicts to declare.

## Supplementary Material

RA-OLF-D6RA06623B-s001

## Data Availability

The data supporting this article, the input settings, the relaxed structures in CIF and POSCAR form, the parsed energies, band, parity, and topology data, and the analysis and plotting scripts, are collected in the accompanying repository at https://doi.org/10.5281/zenodo.21453807, which resolves to the most recent version of the deposit. Licensed pseudopotential files are not redistributed; we give the potential labels, the code version, and input checksums instead. Supplementary information (SI): the plane-wave cutoff and k-mesh convergence tests, Fu-Kane parity counts for the stanene benchmark and free plumbene, the frozen-geometry, Pb/stanene commensuration and buckling-isolation controls, the electronic structure of every stacking registry, the biaxial strain series, HSE06 + SOC gaps, layer-resolved densities of states at the Fermi level, the graphene-interface charge redistribution, Bader charges and two-sided work functions, the direct Pb/hBN Wilson-loop topology with the legacy separation scan, residual phonon minima and 300 K molecular-dynamics stability data, and the energy–strain curves used for the in-plane elastic constants (Tables S1–S13 and Fig. S1). See DOI: https://doi.org/10.1039/d6ra06623b.
