# Supplementary material for: Substrate control of the electronic phase of low-buckled plumbene
Source: RSC Adv. 2026 Sep 8;16(43):53365–74. doi: 10.1039/d6ra06623b (PMC13551297; doi:10.1039/d6ra06623b)
Supplement: Supplementary file 1 [file RA-016-D6RA06623B-s001.pdf]

Supplementary Information (SI) for RSC Advances.
This journal is © The Royal Society of Chemistry 2026

Electronic Supplementary Information

Substrate control of the electronic phase of low-buckled plumbene

Nico Simoni and Daniel Hashemi

Department of Physics, Optical Engineering, & NanoEngineering, Rose-Hulman Institute of Technology, Terre Haute, Indiana 47803, USA. E-mail: hashemi@rose-hulman.edu

S1. Convergence

Total energy per atom against the plane-wave cutoff drifts by a few meV over 400 to 600 eV; the working cutoff is 500 eV.

Table S1. Total energy per atom (eV) against the plane-wave cutoff ENCUT for free plumbene, graphene, and hBN.

ENCUT (eV)	plumbene	graphene	hBN
400	-3.002	-9.232	-8.797
500	-3.002	-9.224	-8.800
600	-3.002	-9.227	-8.802

Energies in eV per atom. The initial non-SOC weak-binding landscape was checked by recomputing every graphene and hBN registry at a 600 eV cutoff and on a 21×21 mesh (data/registry_energy_convergence.csv). The definitive graphene ordering and phases use the stricter full-SOC relaxations and matched SOC statics reported in S4a, not this initial scan.

Table S2. Initial non-SOC registry energy differences ΔE (meV per cell) for Pb/graphene and Pb/hBN at three cutoff and k-mesh settings, referenced to the hollow registry.

partner	registry	ΔE (meV), 500 eV / 15×15	600 eV / 15×15	500 eV / 21×21
graphene	hollow	0.00	0.00	0.00
graphene	top	6.11	6.08	6.43
graphene	bridge	6.36	6.32	6.86
hBN	hollow (ground state)	0.00	0.00	0.00
hBN	over N	1.29	1.62	1.45
hBN	over B	15.77	15.73	15.45

Each initial non-SOC difference changes by at most 0.5 meV per cell across these settings. The closest pair (hBN hollow versus over-N, 1.3 to 1.6 meV) is consistently ordered but

near-degenerate, which is the basis for describing those registries as nearly free-sliding. Band gaps and band-edge locations were extracted on a 21×21 mesh; the fundamental gap of free plumbene changes by less than 5 meV between 15×15 and 21×21.

S2. Topological index by the Fu-Kane parity criterion

Z_2 of the gapped, centrosymmetric systems is the product over the four time-reversal-invariant momenta (TRIM) of the parity products of the occupied bands. The workflow is the standard one: the spin-orbit charge density is converged self-consistently on a full-zone 15×15 Γ-centred mesh; the four TRIM are then computed non-self-consistently from that fixed density (ICHARG = 11); and the parities are read from the resulting wavefunctions with irrep at the full plane-wave cutoff, after shifting the cell so the inversion centre sits at the origin. The counts are unchanged when the wavefunction-reconstruction cutoff is varied from 100 to 500 eV (data/parity_ecut_convergence.csv), every state returns a clean ±1 parity, and the benchmark reproduces the known nontrivial index of stanene. (An earlier pass had converged the density at the TRIM themselves; redoing every system with the full-zone protocol reproduced identical parity counts, so both are deposited histories of the same result, with the full-zone set the one of record.) The parity counts are read directly from the deposited wavefunctions by scripts/parity_from_wavecar.py (clean completion, raw JSON per system in data/parity_raw/).

Table S3. Fu-Kane parity counts at the four time-reversal-invariant momenta and the resulting Z_2 index for stanene (benchmark) and free plumbene.

System	Γ	M_1	M_2	M_3	δ(Γ,M,M,M)	Z_2
stanene (benchmark)	12	14	14	14	+, −, −, −	1
free plumbene	14	14	14	14	−, −, −, −	0

Entries are the number of parity-odd occupied states; δ = (−1)^(n_odd/2); Z_2 is nontrivial when the product of the four δ is −1. Stanene returns the correct nontrivial index; plumbene is trivial, differing only at the zone centre.

S3. Frozen-geometry control (electronic versus structural metallisation)

The plumbene layer, extracted from each relaxed bilayer at its exact coordinates and cell, computed in isolation. The structures used are the relaxed bilayer geometries deposited with this ESI.

Table S4. Frozen-geometry control: the plumbene layer computed in isolation at the exact coordinates and cell of each relaxed bilayer.

isolated plumbene layer	Pb buckling (Å)	fundamental gap (eV)	verdict
free (reference)	0.92	+0.42	insulator
from Pb/graphene (hollow)	0.97	+0.375	insulating alone; interface overlap is electronic
from Pb/hBN	0.93	+0.43	insulator

isolated plumbene layer	Pb buckling (Å)	fundamental gap (eV)	verdict
from Pb/stanene	1.42	−0.74	already metallic: bilayer metal is structural

S4. Pb/stanene commensuration and buckling controls

The stanene metallisation does not depend on how the 5.6% lattice mismatch is partitioned between the layers, and traces to the buckling itself. All four rows are coherent 1x1 Pb/Sn cells at a different imposed common lattice constant; they probe the strain partitioning, not a larger coincidence supercell or a rotated moiré, which the 5.6% mismatch would require and which we do not test here.

Table S5. Coherent 1×1 Pb/stanene cells at different imposed common lattice constants: Pb strain, Pb buckling, fundamental gap, and phase (strain-partitioning control).

common a (Å)	Pb strain	Pb buckling (Å)	fundamental gap (eV)	phase
4.937 (plumbene's own, Pb unstrained)	0.0%	1.28	−0.47	metal
4.800 (intermediate 1x1)	−2.8%	1.42	metallic	metal
4.674 (stanene's own)	−5.3%	1.47	−0.87	metal
4.635 (energy-optimal)	−6.1%	1.50	−0.95	metal

Buckling-isolation, free plumbene at its own lattice with the buckling imposed:

Table S6. Buckling-isolation control: free plumbene at its own lattice constant with the equilibrium and the stanene-interface buckling imposed.

imposed buckling (Å)	fundamental gap (eV)	phase
0.92 (equilibrium)	+0.42	insulator
1.42 (stanene value)	−0.55	metal

The lead layer buckles and metallises at every common lattice constant, including the row with plumbene unstrained (the strongest control, since the lead layer carries no strain there); and imposing the stanene buckling on the free sheet closes the gap on its own. The "energy-optimal" cell is taken from a variable-cell relaxation and is included only to bracket the range; the metallic outcome does not rely on it.

S4a. Stacking-registry electronic structure

To determine both the stacking order and the electronic phase without relying on non-SOC geometries, we independently relaxed all three Pb/graphene registries with SOC below 0.001 eV $Å^{-1}$ and then computed their SOC electronic structures on the same dense 21×21×1 mesh. Because their energy separations are on the meV scale, every final geometry was additionally recomputed at 600 eV/21×21×1 and 500 eV/30×30×1; the ground registry was accepted only if all three protocols agreed above a 1 meV-per-cell

numerical margin (`graphene_SOC_registry.csv` and `graphene_SOC_registry_convergence.csv`). The stanene and hBN rows below retain the original all-registry comparison.

Table S7. Pb buckling, fundamental gap, and electronic phase of every Pb/stanene, Pb/graphene, and Pb/hBN stacking registry (PBE+SOC); the graphene rows are the independently SOC-relaxed registries.

partner	registry	Pb buckling (Å)	fundamental gap (eV)	phase
stanene	AA (ground state)	1.39	−0.61	metal
stanene	bridge	1.14	−0.31	metal
stanene	AB	1.32	−0.35	metal
graphene	hollow (ground state)	0.974	−0.076	semimetal (indirect overlap)
graphene	bridge	0.974	−0.074	semimetal (indirect overlap)
graphene	top	0.974	−0.073	semimetal (indirect overlap)
hBN	hollow (ground state)	0.88	+0.323	insulator
hBN	over N	0.87	+0.372	insulator
hBN	over B	0.88	+0.369	insulator

Every stanene registry is a robust metal (including the weakly bound AB registry, which binds at roughly half the AA energy yet still buckles the lead layer to 1.32 Å), and every boron nitride registry is an insulator. Every independently SOC-relaxed graphene registry is instead a semimetal with a negative indirect gap and a positive minimum direct gap (0.197–0.203 eV). Hollow is the graphene ground registry in all three numerical protocols. Its margins over the next registry are 3.045, 3.028, and 3.275 meV per cell for 500 eV/21×21×1, 600 eV/21×21×1, and 500 eV/30×30×1, respectively; top and bridge remain mutually near-degenerate. This common ordering above the predeclared 1 meV gate and the common semimetallic phase remove the non-SOC-registry ambiguity discussed by Referee 1.

S4b. Pb s/p-resolved band character

The orbital-resolved bands in Fig. 3 of the main text use the site- and angular-momentum projections written by VASP for the PBE+SOC band paths. Pb-s weight and the sum of Pb-px, Pb-py, and Pb-pz weights are shown separately; all partner-orbital and interstitial weight is left uncoloured. The numerical data are provided as `free_plumbene_pb_sp.csv`, `pb_graphene_pb_sp.csv`, `pb_hbn_pb_sp.csv`, and `pb_stanene_pb_sp.csv`. The bands closest to the free-plumbene gap are Pb-p-dominated. hBN preserves this separated Pb-p manifold, and stanene hybridises its heavy-element manifold with the strongly buckled Pb-

p states. For the accepted hollow Pb/graphene ground, 91.2% of the material Pb-s/Pb-p projection carried by 318 states within ±0.5 eV of E_F is Pb-p weight; the separate layer projection assigns 72.4% of the states within 50 meV to Pb. Pb-s weight is concentrated mainly farther from the Fermi level.

S5. Biaxial strain: fundamental gap and band-edge locations

Internal coordinates relaxed at each fixed strained cell; band extrema from a dense 21×21 mesh.

Table S8. Biaxial strain series of free plumbene: valence- and conduction-band-edge locations, fundamental gap, and Fu-Kane Z_2 index at every gapped strain.

strain	VBM (k)	CBM (k)	fundamental gap (eV)	Z_2
−8%	Γ	K	metallic (−1.213)	n/a
−6%	Γ	K	metallic (−0.539)	n/a
−4%	Γ	K	metallic (−0.044)	n/a
−3.9%	Γ	K	metallic (−0.024)	n/a
−3.8%	near Γ	K	−0.004	n/a
−3.7%	near Γ	K	0.010	0
−3.6%	near Γ	K	0.025	0
−3.5%	near Γ	K	0.036	0
−3%	near Γ	K	0.134	0
−2.5%	near Γ	K	0.221	0
−2%	near Γ	K	0.279	0
0%	K	K	0.418	0
+2%	K	K	0.419	0
+4%	K	K	0.424	0
+6%	K	K	0.430	0
+8%	K	K	0.439	0

Compression closes the gap as an indirect Γ−K overlap. A dense series in 0.1% steps brackets the crossing between −3.7% (a 0.010 eV gap) and −3.9% (a 0.024 eV overlap); the 4 meV overlap at −3.8% is within the numerical resolution of a few meV, so the transition is quoted as an interval, −3.7% to −3.9%. The Z_2 index was computed directly by the Fu-Kane parity criterion (full-zone density, then non-self-consistent TRIM; Section S2) at every gapped strain in the table, including −3.6% and −3.7% inside the transition region, returning n_{odd} = (14, 14, 14, 14) at the four time-reversal-invariant momenta and hence Z_2 = 0 in every case; the raw outputs are in the repository (data/parity_raw/). Independently, the minimum direct gap stays near 0.43 eV throughout the compression range (data/gaps_strain.csv), so no band-gap closure and reopening occurs anywhere on the path to the transition. All strain gaps and band-edge k-locations are in data/gaps_strain.csv.

S6. HSE06+SOC robustness

Self-consistent HSE06+SOC calculations used a 500 eV cutoff and Gamma-centred 6×6×1 meshes containing K at the same fixed geometries used for the PBE+SOC comparison. Free plumbene has a 0.580817 eV direct and fundamental gap at K, compared with 0.418 eV at PBE+SOC. Pb/hBN remains insulating with a 0.492184 eV direct gap at K. Pb/stanene retains gapless indirect character: its fundamental gap is −0.553682 eV, while its minimum direct gap remains positive at 0.380731 eV. The accepted hollow Pb/graphene ground was recomputed from scratch with VASP's symmetry-preserving hybrid-functional setting, zero initial moments, `ISTART=0`, and `ICHARG=2`. It converged to a maximum site moment of 0.0 μ_B, an indirect overlap of −0.056028 eV, and a 0.000793 eV minimum direct gap. The result passed exact symmetry-reduced EIGENVAL/IBZKPT, weighted-electron-count, convergence, stream, provenance, and output-hash gates; it therefore establishes the nonmagnetic semimetallic phase without relying on earlier calculations excluded by the 0.005 μ_B/site acceptance threshold.

Table S9. HSE06+SOC fundamental and minimum direct gaps on Γ-centred 6×6×1 meshes at the fixed PBE+SOC geometries.

System	Fundamental gap (eV)	Minimum direct gap (eV)	HSE06+SOC phase on 6×6×1 mesh
free plumbene	0.580817	0.580817	insulator
Pb/hBN	0.492184	0.492184	insulator
Pb/stanene	−0.553682	0.380731	indirect band overlap
Pb/graphene (hollow)	−0.056028	0.000793	indirect band overlap

The table distinguishes fundamental and minimum direct gaps; the coarser hybrid mesh is disclosed and the comparison is used only for the qualitative phase/gap character it resolves. Parsed extrema, k-point coordinates, final site moments, and acceptance gates are deposited in `data/hse_soc_gaps.csv`.

S7. Layer-resolved density of states at the Fermi level

Fraction of the density of states at E_F carried by the lead layer, from the spin-orbit layer-projected DOS. On Pb/stanene the two layers contribute comparably (lead share near 52%). On Pb/hBN the density of states at E_F is zero (the 0.34 eV gap is intact).

On Pb/graphene the density of states exactly at E_F is sensitive to sampling because the partner Dirac point also lies there. We therefore recomputed the accepted full-SOC ground registry on three meshes that all contain K, using the same 0.05 eV Gaussian width, and report the sensitivity rather than selecting one favourable number:

Table S10. Pb share of the density of states at E_F for the hollow Pb/graphene ground registry on three K-containing k-meshes at fixed Gaussian smearing.

k-mesh	Gaussian smearing (eV)	Pb share of DOS at E_F

k-mesh	Gaussian smearing (eV)	Pb share of DOS at E_F
21×21×1	0.05	0.770634
27×27×1	0.05	0.715163
33×33×1	0.05	0.859882

The Pb share remains between 0.715163 and 0.859882 across the three K-containing meshes (spread 0.144719), so the Pb-majority near-E_F assignment is not selected from one favourable mesh. All reported quantities are density-of-states or band-projection weights, not transport or conductivity claims.

S8. Graphene interface charge redistribution

We formed $\Delta\rho(z)=\rho_{Pb/graphene}-\rho_{Pb}-\rho_{graphene}$ from byte-matched cells and FFT grids. The planar profile spans -1.110547e-03 to 8.360767e-04 e Å^-3; the largest cumulative displaced charge is 0.017792 e and the cell-integrated residual is −0.00000000 e, within the 0.01 e conservation gate. These are redistribution measures rather than an integer-transfer claim; the independent Bader partition gives −0.027 e on the Pb layer.

S9. Charge transfer and two-sided work functions

Bader charge on the plumbene layer (positive = gained), all-electron referenced (LAECHG). Work function on each face of the asymmetric slab.

Table S11. All-electron Bader charge on the Pb layer and the work function on the Pb face and the partner face of each bilayer.

bilayer	charge on Pb layer (e/cell)	Φ, Pb face (eV)	Φ, partner face (eV)
Pb/graphene (hollow)	−0.027	4.40	4.12
Pb/hBN	−0.03	3.85	4.23
Pb/stanene	+0.02	4.42	4.42

For insulating Pb/hBN a Fermi-referenced work function is not unique, because the position of the chemical potential inside the gap is not fixed by the calculation (the tabulated 3.85 eV coincides with the electron affinity because the computational Fermi level sits at the conduction-band edge; it should not be read as an intrinsic work function). The well-defined quantities are: the vacuum-level difference between the two faces, 0.38 eV (partner face higher, the interface dipole step); the ionisation potential V_{vac} − VBM, 4.19 eV on the plumbene face and 4.57 eV on the partner face; and the electron affinity V_{vac} − CBM, 3.85 eV on the plumbene face and 4.23 eV on the partner face. Each pair brackets the 0.34 eV gap.

S10. Direct Pb/hBN Wilson-loop topology

The Pb/hBN index is obtained from the eigenphases of closed Wilson loops built directly from first-principles overlaps of all 60 occupied spinor bands, not from discrete interlayer-distance sampling or a fitted tight-binding Hamiltonian. VASP wrote the occupied-subspace overlaps through its Wannier90 interface, and Z2Pack adaptively refined both points per

loop and transverse lines. The final Pb/hBN surface contains 60 WCCs per line on 16 transverse lines; its position, largest-gap, and motion convergence reports contain no failed or missing entries. The maximum endpoint Kramers-pair splitting is 8.263×10^{-9}, and two crossings of the largest-gap reference give Z_2=0. Under the identical settings, stanene gives Z_2=1 with 27 crossings on 19 lines and free plumbene gives Z_2=0 with two crossings on 13 lines, in agreement with their independent Fu–Kane parity indices. Every final overlap file was checked for electronic convergence, a normal VASP footer, the requested occupied dimension, and complete matrix-record count. Attempts to construct an additional s/p MLWF model were excluded because they did not pass the predeclared formal-localisation and interpolation gates; no unconverged Hamiltonian contributes to the reported index. The legacy separation scan below is retained only as supporting evidence; the interlayer distance is the separation between the mean Pb and mean hBN planes, the same definition used in Table 4 of the main text.

Table S12. Legacy Pb/hBN interlayer-separation scan of the fundamental gap, retained as supporting evidence only.

interlayer d (Å)	fundamental gap (eV)
3.9 (bonded)	0.34
4.5	0.41
5.0	0.43
5.4	0.43
9.9 (isolated)	0.44

S11. Lattice dynamics and thermal stability

Finite-displacement force constants were computed without SOC on the supercells listed below. Phonopy space-group symmetrisation followed by traditional force-constant symmetrisation enforces the translational acoustic sum rule: the maximum force-constant drift is zero to the printed precision after the treatment. The residual minima and eigenvectors are:

Table S13. Residual phonon minima after acoustic-sum-rule enforcement: supercell, minimum frequency, reduced wavevector, out-of-plane eigenvector fraction, and mode character.

system	supercell	minimum (cm^{-1})	reduced q	out-of-plane fraction	mode character
free plumbene	4×4×1	−6.55	(0.1400, 0.0700, 0)	0.807	flexural acoustic
Pb/graphene	3×3×1	−5.66	(0.0000, 0.0000, 0)	0.000007	relative interlayer in-plane shear
Pb/stanene	3×3×1	−7.35	(0.5834, 0.1666, 0)	0.010	in-plane acoustic-like
Pb/hBN	2×2×1	−11.85	(0.2200, 0.1100, 0)	0.798	flexural acoustic

ASR removes translational drift but does not remove these small negative values. The free-plumbene and Pb/hBN minima are predominantly out of plane and are consistent with finite-supercell interpolation of a quadratic flexural branch. Pb/graphene instead has a $-5.66\ cm^{-1}$ minimum at Gamma with an out-of-plane fraction of only 7.3×10^{-6}; the in-plane eigenvectors of the Pb and graphene layers are antiparallel (cosine −1.000), so this is a relative interlayer shear mode. Its small magnitude is compatible with the shallow registry-energy landscape of the weakly bound interface, but it remains a reported soft mode rather than being relabelled as a flexural artefact. Pb/stanene has a small in-plane feature away from Gamma. We therefore report the modes rather than asserting that every negative value is a flexural numerical artefact, and do not claim converged harmonic dynamical stability without larger-supercell tests.

Before molecular dynamics, each canonical unit cell was re-relaxed without SOC to a maximum force below 0.001 eV $Å^{-1}$ using the same PBE or optB86b-vdW functional used in the trajectory and was expanded exactly to a 4×4 free-plumbene or 3×3 bilayer supercell. Trajectories used `PREC = Normal`, a 500 eV cutoff, `EDIFF` = 10^{-5} eV, Gaussian smearing with σ = 0.05 eV, `LREAL = Auto`, `ISYM = 0`, and the blocked-Davidson/RMM-DIIS solver (`ALGO = Fast`) with `NELMIN = 4`; the electronic state was initialised afresh at the start of each run rather than from a stored charge density or wavefunction. Free plumbene and Pb/stanene used `NELM = 200` with default mixing and band counts. Pb/graphene and Pb/hBN used `NELM = 500`, damped linear mixing (`AMIX` = 0.1, `BMIX` = 10^{-4}, `AMIN` = 0.01), and `NBANDS = 312` (resolved to 320 by the 16 band groups of the 128-rank runs): initial production attempts on both systems failed with an identical RMM-DIIS subspace-diagonalisation (ZHEGV) collapse whose wavefunction-orthogonality warnings were confined to the highest empty bands (327–336 of 336), and removing those bands eliminated the failure. The physical protocol tags (thermostat, time step, target temperature, cutoff, `EDIFF`, smearing, k-sampling) are identical across the four systems, and the solver-robustness tags cannot change the converged result, only whether it is reached.

Each system was screened before production with paired 48-step trials under two frozen velocity seeds. A system became eligible for production only if both seeds converged the electronic loop at every ionic step and the measured cost projected within the allocation; the production run then replayed the second seed from ionic step 1. All accepted productions used a Nosé-Hoover thermostat, a 2 fs time step, 1500 steps, and a 300 K target. Every ionic step of an accepted trajectory carries the `EDIFF` convergence marker, and the captured standard output, OUTCAR, and scheduler standard error were required to contain no electronic non-convergence or internal VASP failure. No screening trajectory or partial segment was reused, and no prefix was concatenated into a production run. free plumbene averaged 299.9 ± 12.1 K, with an extended-system-energy drift of −0.170 meV atom^-1 ps^-1 and a Pb-buckling range of 0.75–0.95 Å; it retained every initial bond and formed no new bond. Pb/graphene averaged 294.9 ± 37.8 K, with an extended-system-energy drift of −1.086 meV atom^-1 ps^-1 and a Pb-buckling range of 0.03–0.92 Å; its minimum mean-plane separation was 3.45 Å (88.4% of the initial value); it broke 5 and formed 20 bond(s), retaining a minimum fraction of 0.815 of its initial bond network. Pb/stanene averaged 299.9 ± 18.7 K, with an extended-system-energy drift of −0.234 meV atom^-1 ps^-1 and a Pb-buckling range of 1.27–1.79 Å; its minimum mean-plane

separation was 2.61 Å (79.1% of the initial value); it broke 8 and formed 33 bond(s), retaining a minimum fraction of 0.815 of its initial bond network. Pb/hBN averaged 295.5 ± 57.8 K, with an extended-system-energy drift of −0.211 meV atom^-1 ps^-1 and a Pb-buckling range of 0.00–0.89 Å; its minimum mean-plane separation was 3.84 Å (99.6% of the initial value); it broke 5 and formed 24 bond(s), retaining a minimum fraction of 0.815 of its initial bond network. Connectivity was measured, not gated: Pb/graphene, Pb/stanene and Pb/hBN changed bond network during the run (minimum retained fraction 0.815), while free plumbene retained every initial bond. The substrate sublattices of the rigid substrates were themselves unchanged. This bond rearrangement is a reported result, not a failed acceptance test. The tests establish only short-time finite-cell behaviour under this protocol. The deposited summary gives the mean and standard deviation of temperature, conserved extended-system energy drift per atom, starting-structure displacement, minimum mean-plane separation, fixed-sublattice Pb buckling and height span, and final-versus-initial nearest-neighbour connectivity for each trajectory, together with the strict-relaxation, screening-trial, production-job, all three captured streams, and checksum provenance.

S12. In-plane mechanical properties

The elastic response of free low-buckled plumbene was obtained from biaxial and Cartesian uniaxial strains between −2% and +2% in 0.5% steps. Internal coordinates were relaxed at every fixed cell until the maximum force was below 0.001 eV $Å^{-1}$, followed by a 21×21×1 static calculation. For a hexagonal sheet the fitted energy densities are U_biax=(C11+C12)ε^2 and U_uniax=C11 ε^2/2. The fits (R^2 = 0.99952 and 0.99992 for biaxial and uniaxial strain, respectively) give C11=17.15 N m^{-1} and C12=11.10 N m^{-1}, hence C66=(C11−C12)/2=3.03 N m^{-1}, Y2D=$(C_{11}{}^2-C_{12}{}^2)/C_{11}$=9.97 N m^{-1}, and ν=C12/C11=0.647. The two-dimensional Born criteria C11>|C12| and C66>0 are satisfied, establishing elastic stability against infinitesimal in-plane deformations within the low-buckled local minimum. The full energy–strain table, fit residuals, and plot are deposited; the fitted curves are shown in Fig. S1.

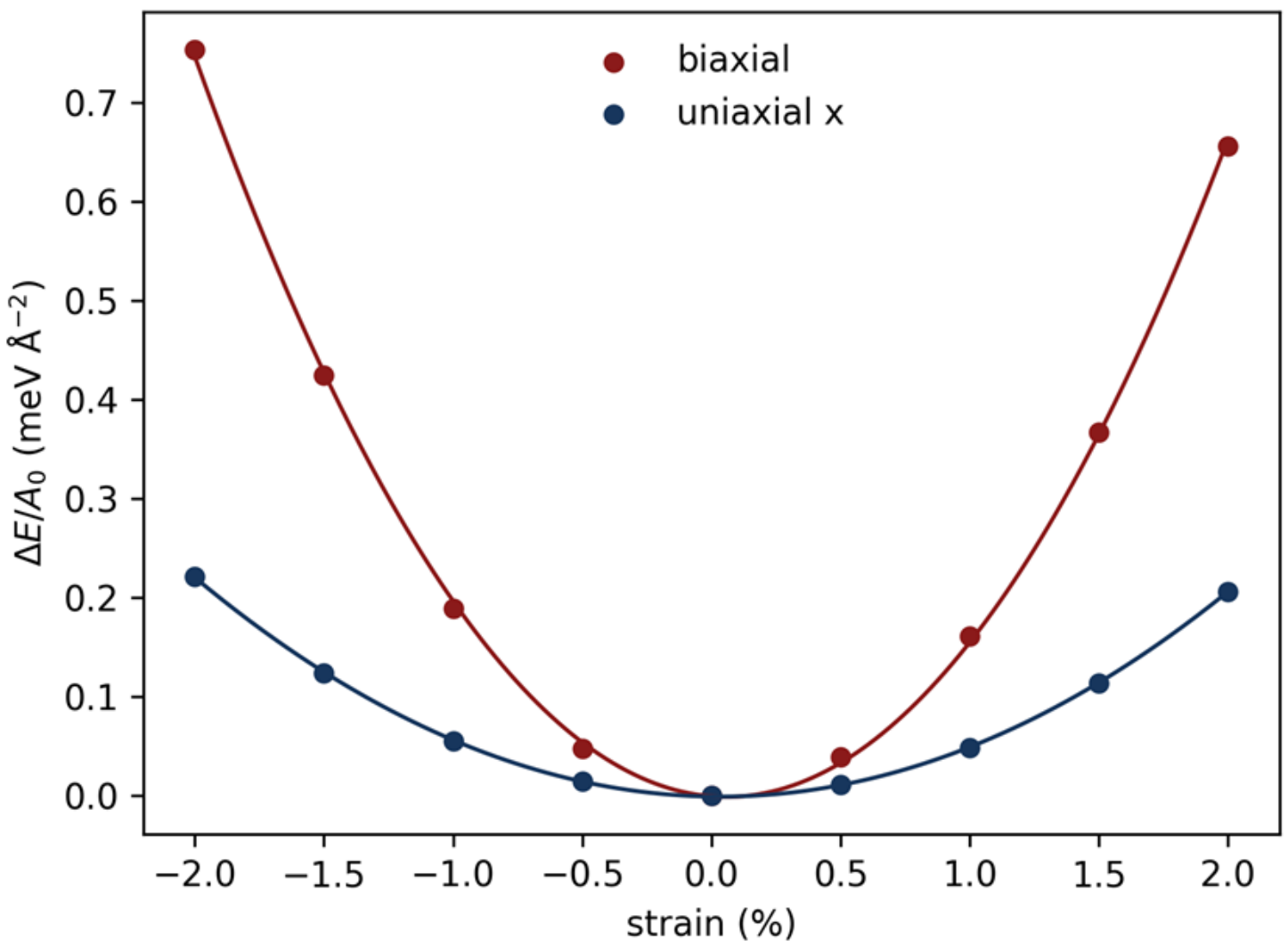

Fig. S1. Biaxial and Cartesian-uniaxial energy density of free low-buckled plumbene over −2% to +2% strain. Symbols are calculated static energies after internal relaxation and lines are quadratic fits.

S13. Data availability

The accompanying repository contains: relaxed structures for every reported system in both CIF and POSCAR form; parsed band-edge gaps and k-locations for the full strain series (gaps_strain.csv) and the three bilayers (gaps_bilayers.csv); the raw all-electron Bader ACF.dat files and net Pb charges (bader/); the per-time-reversal-invariant-momentum parities with recomputed Z_2 and the raw irrep outputs (parity_Z2.csv, parity_raw/); the layer-resolved density of states at the Fermi level as a function of k-mesh and smearing (dos_layer_fraction.csv); the electronic structure of every stacking registry; the planar charge-density-difference profile and its cumulative integral (cdd_planar_profile.dat, cdd_cumulative_Q.dat); final ASR phonon band.yaml files with eigenvectors and FORCE_SETS files (phonon_asr/); the hBN interlayer-distance scan (hbn_continuity.csv); and redistributable input decks without POTCAR. The individual analysis scripts re-derive the new tables and figures from those artifacts, while scripts/validate_package.py checks the expected systems, convergence/acceptance flags, numerical thresholds, linked figures, document builds, and absence of licensed pseudopotentials. The per-trajectory AIMD inputs and raw dynamics streams (INCAR, KPOINTS, POSCAR, CONTCAR, OSZICAR, XDATCAR) and the phonon FORCE_SETS files are provided in a companion archive under the same DOI; the heavier density and wavefunction outputs (OUTCAR, EIGENVAL, PROCAR, CHGCAR, AECCAR) are retained in the archived cluster deposit and are available from the authors on request. Licensed pseudopotential files are not redistributed; potential labels, VASP versions (6.6.0 and 6.6.1, with the allocation VASP 6.6.1 executable linked to Wannier90 3.1.0 for overlap output), and input checksums are provided instead.
